# Towards an Understanding of Population Health Data in a Single NHS Trust during COVID-19

**DOI:** 10.3390/healthcare10030447

**Published:** 2022-02-26

**Authors:** Sally Fowler Davis, Simon Choppin, Shona Kelly

**Affiliations:** Advanced Wellbeing Research Centre, Sheffield Hallam University, Sheffield S1 1WB, UK; s.choppin@shu.ac.uk (S.C.); s.kelly@shu.ac.uk (S.K.)

**Keywords:** COVID-19, communicating population data, inequalities data visualisation

## Abstract

The objective of this study was to determine the further care needs of people discharged from the hospital following a COVID-19 illness from April–September 2020. Methods: In partnership with an NHS trust in the UK, data analysis was undertaken by linking data from the Trust, to facilitated a triage process. The intention was to provide information in a format that enabled an examination of the population data and highlight any inequality in provision. Data were mapped onto the indices of multiple deprivation, and a range of text and graphical methods were used to represent the population data to the hospital leadership. The visual representation of the demographics and deprivation of people discharged during a critical period of the pandemic was intended to support planning for community services. The results demonstrated that just under half of those discharged were from the poorest fifth of the English population and that just under half were aged 75 or older. This reflected the disproportional effect of COVID-19 on those who were poorer, older or had pre-existing multiple morbidities. Referral to community or outpatient services was informed by the analysis, and further understanding of the diversity of the population health was established in the Trust. Conclusion: By identifying the population and mapping to the IMD, it was possible to show that over half of discharged patients were from deprived communities, and there was significant organisational learning bout using data to identify inequalities.. The challenge of planning services that target underserved communities remains an important issue following the pandemic, and lessons learnt from one health system are being shared.

## 1. Introduction

The COVID-19 pandemic has demonstrated the ability of healthcare systems to maintain acute services and respond to urgent healthcare needs in the face of extreme pressure, but healthcare system recovery and future resilience has been called into question [1] with a need to address the under-resourcing of staff and the digital upgrade across services. Furthermore, COVID-19 exposed systematic health inequalities [2] based on poverty and pre-existing ill health and multiple morbidity.

A key consideration at the early stage of the pandemic (between April and September 2020) was whom to prioritise for acute admission, with the hospitalisation of those with severe illness and the subsequent reduction in access to elective care [3]. Subsequently, patients were discharged, and further analysis of community care was needed so that follow-on rehabilitation needs could be assessed on discharge. In recognition of treatment pathways for specific conditions and clinical specialities, COVID-19 was categorised within several medical specialisms, including infectious diseases, respiratory diseases, neurology services and ICU, with their associated outpatient clinics. However, it quickly became clear that patients experienced longer-term symptoms and had continuing needs that could be managed in community services [4]. There was a need to justify a new service specification based on the follow-on needs of those suffering relapsing and remitting symptoms post-COVID [5]. Narrative reports from patients suggested that pain, fatigue and breathlessness continued after 12 weeks of recovery [6] and that there were additional psychological difficulties, in part associated with their acute hospitalisation, and for others a response to lockdown, de-conditioning and isolation [7].

Managers in health and social care needed to access population data [8] to reflect the variation in patient age, location and further rehabilitation requirements. They sought to enable stakeholder engagement and decision making across acute (medical) and multidisciplinary community services in order to design a new pathway of care for this patient cohort based on a city-wide requirement. Previous work by Kithin and McArdle (2016) focused on city-wide planning [9]; they also reported that ‘dashboards’ are useful tools to evaluate and manage services and fundamentally support knowledge exchange across diverse and multi-disciplinary groups [10]. However, there are few other examples of how real-time data have been used to inform critical and timely decision making at the city level (see [11,12]), where population health data are used to inform community services. We also were working with a service that had not previously used data to inform decisions, so there was a steep learning curve.

The aim of this study was to determine the needs of people discharged from the hospital following a COVID-19 admission. It was based on a partnership between our university and an NHS trust which evaluated the outcomes of a triage process undertaken by the trust. The objectives were to (a) present some of the demographic data for those admitted and discharged from the hospital and (b) describe the results of the data analysis to healthcare leaders who were planning further services. In addition, a reflection on the methods of sharing data and data visualisation is included to support the ongoing data management that benefits decision making associated with service planning for long COVID.

## 2. Methods

Using an evaluation approach to the systematic analysis of population health data [13], a simple design was agreed upon with the NHS trust as the sponsoring organisation. Whilst there was no a priori theoretical assumption about who was being treated in hospitals (as opposed to in community services or primary care), there was a concern that social and economic determinants of health may be a factor for patients in accessing the need for hospital and follow-up services [14]. The selection of priorities for the evaluation was based on feedback and participation at improvement planning meetings within the trust. Leaders were familiar with microsystems improvement methods and iterative planning [15]. The data and discussion were used to inform decision making about the ongoing and rapidly changing situation with regard to service need.

### 2.1. Approval

The approval for the project was received via NHS trust ‘Silver Command’ and was based on the data governance mechanisms in place during the COVID-19 pandemic, i.e., Control of Patient Information (COPI) regulations (to end September 2020). This permitted the sharing of anonymised data for developmental reasons [16] and was confirmed by e-mail by the Data Protection Officer [17]. No identifiable data were exported for the purpose of analysis given that the SPA was responsible for community service referral and the academic analysis was for the purpose of data analytics and visualisation, allowing NHS leaders to gain insight into the demographic spread of the population admitted with COVID-19 and discharged into community services.

### 2.2. Data Linkage

Four datasets were compared and analysed for the purpose of the evaluation. Firstly, routine data were collected by the NHS trust from electronic patient record (EPR) systems, namely Lorenzo and ‘White Board’. Secondly, data for outpatients, inpatients and intensive care units (ICU) were derived from Secondary Uses Service (SUS) data, and thirdly, community services were collected from SystmOne—an alternative EPR system used by community and primary care services. For the first time, the NHS Trust Information Service undertook to link the datasets for the purpose of identifying all those who were discharged into the community following a ‘COVID admission’. Data from each of the electronic record systems were linked using a project-specific pseudonymised ID number. The data were collated by the internal data analyst in SPA, and the triage data were shared anonymously with the university and linked with the cohort care data via the project-specific pseudonymised ID number.

### 2.3. Triage Process

A second dataset was collected through a triage process, designed by the NHS trust for the purpose of contacting discharged COVID patients and asking them about follow-up needs. This was undertaken by the Single Point of Access (SPA—a patient referral service). The telephone triage tool was co-developed by NHS managers in community services with input from the academic collaborators and was undertaken by telephone contact with patients who had been discharged at any point after 31 March 2020. The triage requested further information on self-reported needs following discharge and measured health-related quality of life using the EUROQOL [15]. This scale asks respondents to rate their health on the current day with 5 levels of severity in 5 domains: usual activity; pain and discomfort; mobility; anxiety and depression; and self-care. There is also a visual analogue scale that asks respondents to select a score out of 100 between anchor points labelled ‘The best health you can imagine’ and ‘The worst health you can imagine’. The selection of this tool was based on its widespread adoption and focus on functional indicators and ease of administration.

### 2.4. Data Analysis

Analysis of the data took place between June and September 2020. Data were made available to the university using CSV-formatted spreadsheets that were converted to SPSS for analysis. Data were transferred by encrypted memory stick and downloaded directly into a secure drive on university systems. The databases provided: age at admission; gender; ethnicity; postcode (used to measure deprivation); a report of death; admission from, or discharge to, a care home; stays in ITU; inpatient stays including the length of each stay; referral to community services and/or outpatient services.

To measure deprivation, the English Indices of Multiple Deprivation (IMD) [18] provided a framework for analysis and measurement of deprivation as a multi-dimensional measure [8]. Conceptually, the IMD provides an integrated measure of advantage or disadvantage and is generated from routinely collected data that are updated regularly. The IMD measure covers seven domains—income; employment; education, skills and training; health and disability; crime; barriers to housing services; and living environment—as key indicators of advantage or disadvantage [19]. The IMD enables the identification of small-area concentrations of deprivation based on a score attributed to the census lower layer super output area (LSOA) geographical equivalent to approximately 1500 persons. The IMD have been mapped onto postcodes to allow for the ranking of individuals into deprivation deciles. In this case, the IMD deciles were reduced into five levels of deprivation to investigate variation in the use of hospital community and outpatient services.

Three other demographic measures were analysed: age, sex and ethnicity. Age categories were selected to divide the original cohort into approximate thirds so that counts were large enough when the data were further stratified. The age categories are consistent with the pattern of increasing risk seen with age: 17–44, 55–74, 75+. Ethnicity was recorded at various points over the past few decades with different choices being available at different points. The concern at the time of the first wave was that people of black ethnicity were at increased risk, but there were relatively few people in the 14 non-white categories in this cohort. Ethnicity was recoded into 4 categories: any mention of Asian (labelled Asian) which includes Bangladeshi, any other Asian background, Chinese, Indian, mixed white and Asian, Pakistani; any mention of black (labelled black) which included any other black background, black African, black Caribbean, mixed white and black African, mixed white and black Caribbean; white only (labelled white) which included any other white background, white British, white Irish; and unknown/mixed which included any other ethnic group, any other mixed background, not known, not stated. Further details including the counts in each ethnic category can be found in the Appendix A.

The findings were presented and shared with managers using predominantly text-based and graphical representations. These were presented alongside discussions and knowledge exchange processes which were used to share insights and understanding of the rapidly changing situation at that time.

## 3. Results

The cohort consisted of all patients who were discharged with a hospital record of having COVID at admission or contracted in the hospital between 30th March and 30th September 2020. The discharged cohort consisted of 1425 patients based on the linked dataset. To be eligible for triage, the patients: had not died after discharge (this was checked each day before attempting contact); were not discharged to a care home (*n* = 342); were not still an inpatient (*n* = 19); and had not had a zero length of stay. At the beginning of the pandemic, patients had to be admitted to be tested for COVID-19 to confirm infection (*n* = 94), and there was a pattern of admitting patients from the city region, from where they were not eligible for community services. One criterion for inclusion was living in the area of the city. The total of the group living within the Sheffield CCG region was *n* = 1247. The other criteria excluded 759 patients (more than one exclusion criteria may apply), leaving 666 patients (Table 1 Column 2). The establishment of the contact list for triage was not a smooth process, and ultimately only 196 were included in the triage data (see Table 1 Column 3) once other patients including those discharged to care homes were excluded.

The results of the analysis are presented below along with some of the challenges associated with data collation and transfer. This data analysis provides an example of how the university supported the information in the services and used it to understand and make early decisions about how to plan further services.

### 3.1. Demographic Analysis

Of the discharged cohort (see Table 1, Column 1), just over three quarters were white and 54% were male. Just under one half were from the poorest fifth of the English population, which is greater than the proportion in the local population. Eighty-five patients could not be assigned to a deprivation pentile, some of whom may have been visitors to the UK. Just under one-half were aged 75 or older with one sixth being admitted from a care home, and just over a quarter died before the triage process was started. Eighty-seven percent had only one inpatient stay, with half staying 7 days or less before discharge or death. Half of the cohort was referred for community or outpatient services.

**Figure 1:**
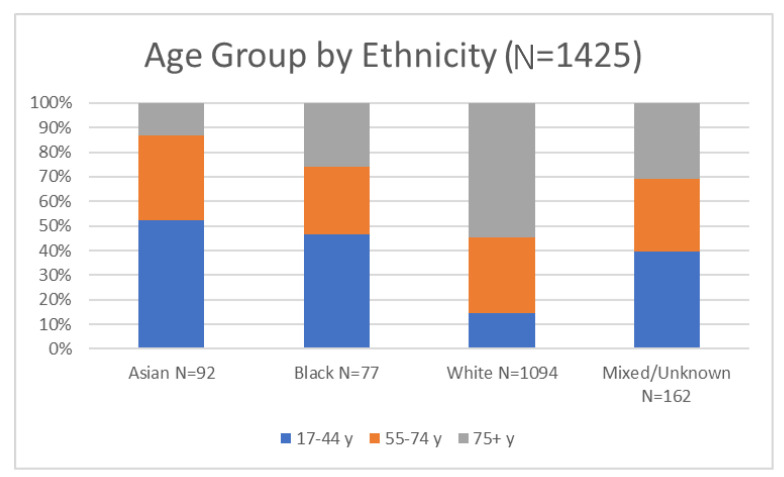
Graph reporting the correlation of age groups by ethnicity (*n* = 1425).

**Figure 2:**
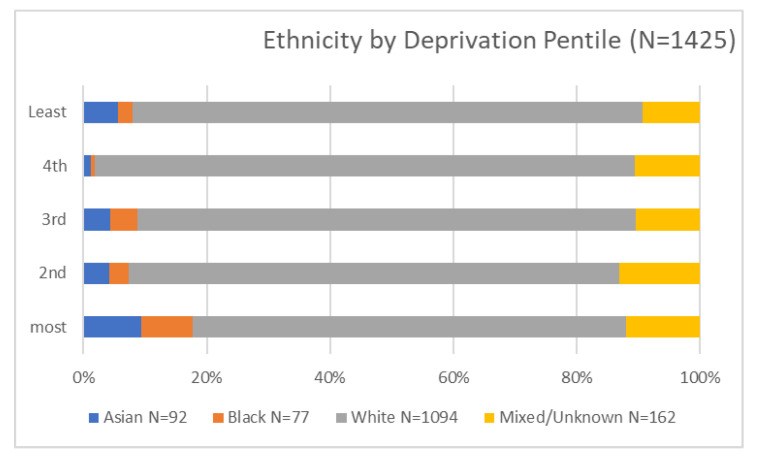
Graph reporting ethnicity of patients by deprivation percentile in IMD.

Amongst those eligible for triage (Column 2 in Table 1), there were proportionately more people eligible for triage from diverse ethnicity than in the original cohort (there are a number of possible reasons which may be linked to deprivation in the area this hospital serves) (see Figure 1). The most deprived make up half of this cohort, and there are distinct differences between pentiles in the proportion on the triage list (see Figure 2). This may be explained by differences in the age profile across pentiles as the young (17–44) were the least likely to go on the triage list (as stated, there were unclear reasons for this within the project rationale). There are no differences in the proportion recommended for triage by length of inpatient stay, and one third were not referred to any community directorate services. A stay in ITU is usually regarded as an indication of disease severity, but in this case and during the early stages of the pandemic, ethnicity appeared to be a strong indicator for admission to ITU, and it is important to note that patients with diverse ethnicity were more often transferred to ITU, perhaps due to more severe symptoms. The supplementary material includes a detailed breakdown of the ethnic codes that were used to identify diverse population groups and shows the small numbers involved in the reporting. Two ways of classifying ethnicity were explored, and this revealed the limitations of the coding and accuracy of the actual ethnic background which was important in relation to (a) a majority group of white, poor patients and (b) the small but diverse needs of different ethnic groups.

The patients who started the triage process (Table 1, Column 3) are not representative of those whom we identified as eligible, but we were unable to resolve the discrepancy. Both the IT and healthcare staff were not used to using or looking at demographic data. We looked for systematic differences in who was included on the triage list based on length of stay or referrals to outpatient services but could not see any systematic source of bias. On the positive side, there did not seem to be any large, systematic demographic differences regarding which patients completed the triage process (Table 1, Column 4), except that completion was greater in people in the two worst deprivation pentiles and was less frequent in patients over age 75 and those with more inpatient stays.

### 3.2. Graphical Representation

Figure 1 and Figure 2 below were presented to NHS trust leaders to represent the total number of patients discharged and across ethnic groups. The cross-tabulation of age and ethnicity was an important factor alongside the rhetoric associated with admissions to ICU (above). In an ethnically diverse city, the disproportional number of white patients in the admitted group, together with a recognition of just under half being over 75, was an important perspective for community service planners.

The proportion of patients from each of the five levels of deprivation is shown, with ‘least’ representing the wealthiest and most advantaged and ‘worst’ being the most deprived. Notably, the most deprived are characterised by larger groups of Asian and black ethnic minorities along with the largest group of mixed or unknown ethnic groups, comprising people who are often white but who may be, for example, Irish or Roma Slovak. The images were useful in relation to orientating leaders to the cohort and beginning to characterise the range of those across different segmented population groups and economic determinants.

### 3.3. Effectiveness of Triage Process

**Figure 3:**
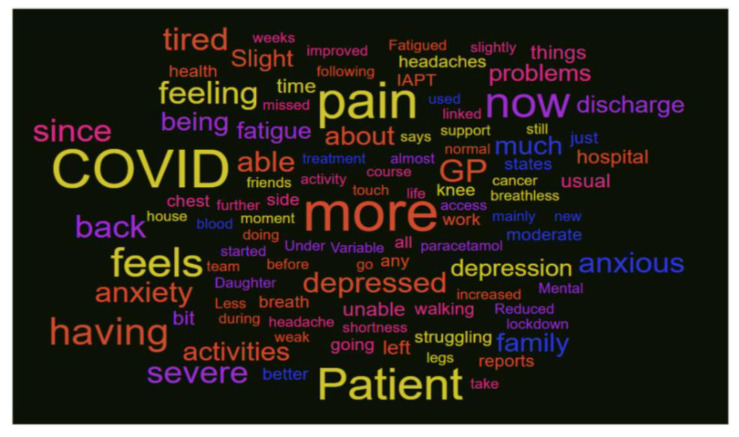
Wordle image combining analysis of triage information reporting primary symptoms.

The triage process was initiated by community services, and patients were contacted by telephone at home with the aim of identifying patients with continuing needs who were continuing to experience symptoms associated with the post-COVID period or long COVID. Figure 3 was used as a means of representing key perceptions of ‘what matters’ to those patients engaging with the triage in the ‘Single Point of Access’ (SPA) workforce. From a clinical perspective, the words to note (and which were highlighted in the presentation) were anxiety/anxious, depression/depressed, pain, (shortness of) breath/breathlessness and tired/fatigue. These words were used in addition to reflections on the individual responses to specific questions in the triage form that indicated follow-on care was needed. The questions and feedback data were used to refine the process of triage and enable triage improvements.

Twenty-nine patients contacted in the triage process refused to be triaged, but there are too few to stratify by demographics. By reviewing the comments noted on the data collection form, the service began to capture (via text analysis) and reflect the dominant reasons. These consisted of triage process errors, full recovery, or a family report that the patient was unavailable or did not need further services. In other cases, the feedback suggested that work commitments, language barriers or sensory deprivation (telephone deafness) were reasons for non-participation.

### 3.4. Results of the Triage Data Analysis

**Figure 4:**
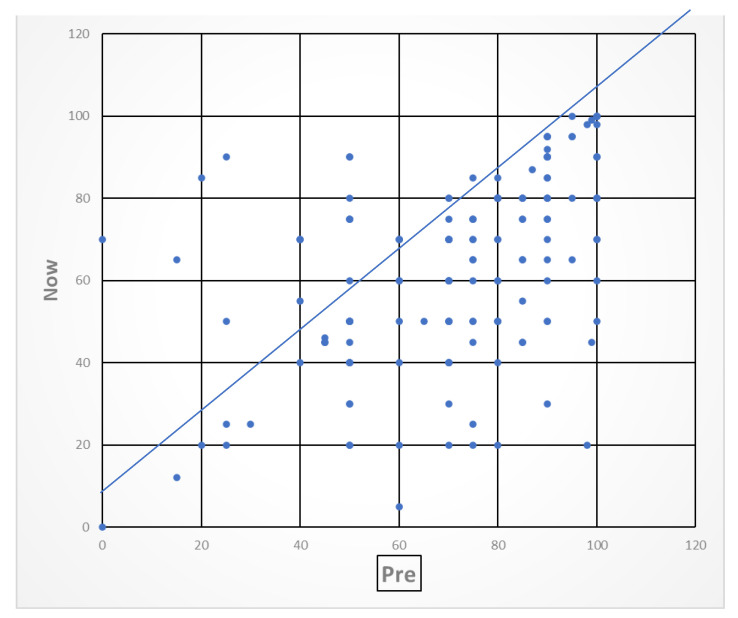
VAS pre-COVID compared with time of interview. Note: The line represents no change in score, and people above the line have better health when triaged compared to before COVID; people below the line are worse.

**Figure 5:**
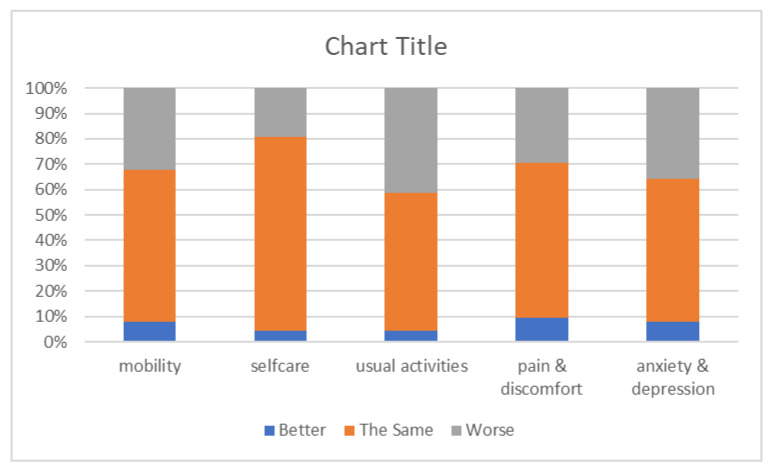
Change in EQ-5D domains reported for before COVID and at time of interview.

The triage responses were analysed and presented in graphical form to enable participating staff and managers to obtain an oversight of early population level needs post-COVID and the indications of the types of community services that were necessary. EQ-5D quality of life VAS scores reported before and after COVID illness at the time of the triage interview demonstrated that the majority of people reported poorer health after discharge than before COVID (see Figure 4). A minority of patients had better VAS scores after compared to before their hospitalisation with COVID, which is not usual as VAS scores are a composite of physical, mental and social health, and few people were systematically worse on all domain scores. Although the individual domain scores were more variable, two-fifths of respondents reported a decline in usual activities. Approximately one third were worse in anxiety and depression, mobility, and pain and discomfort (see Figure 5). There were too few patients in non-white ethnicity groups or better deprivation pentiles to conduct any further analysis by demographics.

A total of 85% of the patients who started the triage process completed it, with approximately 85% of these patients referred to community or outpatient services (Table 1, Column 4). People in the two worst deprivation pentiles were slightly less likely to complete the triage process; those in the top two pentiles were more likely to complete the triage; and females were more likely to complete it than males. The proportion completing the triage declined substantially by age from 93% in those 17–44 years old to 75% in those age 75+. Ninety-six percent of triage patients with a stay in ITU completed the triage process.

### 3.5. Data Limitations and Engagement

The data linkage and analysis took place early in the pandemic using a new method for the trust, and much was done in a short timeframe to enable the data to inform an understanding of the population’s needs. Several rounds of data management were undertaken with involvement between leaders and academic partners. The data analysis was a novel method of sharing the wider population’s needs with acute care staff and community rehabilitation services. In addition to the data selected as examples here, the data were plotted to ‘heatmaps’ that represented each patient with their home address across the city geography. These images were powerful representations of the most affected neighbourhoods but have not been included to maintain anonymity.

## 4. Discussion

The results of the evaluation demonstrated that around half of the total cohort admitted and discharged during the first phase of the pandemic were over 75 and were predominantly white. However, patients from diverse ethnicities were more likely to be admitted to the ICU, as it was an admission criterion, and they were also more likely to be from the poorest neighbourhoods. The triage process was limited by an unclear eligibility criterion, but text-based information suggested the anxiety and pain associated with the post-discharge experience and the standardised tool (EQ5D) reported overall poorer health experience after discharge than before COVID. The quality-of-life domains are highly validated and denote the population’s experience, i.e., very few patients reported that their quality of life had improved in all domains, but the majority (who had recovered from COVID and who had been discharged) felt the same, suggesting that many patients did not need follow-up support, which is important in relation to service planning. It is reasonable to suggest that the older population may have more difficulties relating to ‘usual activity’ and reduced mobility post-admission. Many older patients were discharged to care homes and thus excluded from this study, and the 17–44 group was under-represented in the triage group, so perceptions were mainly for those (a) who could be contacted and (b) were able to report ongoing needs.

### 4.1. Timeliness of the Data Analysis

March to September 2020 was a critical time in the course of the pandemic, during which the needs of acutely ill patients were paramount, and there was an unknown trajectory to the disease process. The description of the needs of discharged patients using demographic data, deprivation, and location across the city was a first insight for this workforce into the later effects of the disease. Service re-design is based on the ability of leaders to assess a population’s needs, including the extent and diversity of demand from different sub-groups of patients [20]. The community service leaders were familiar with the management of care for people with long-term conditions, particularly older patients with rehabilitation needs but not those with post-COVID syndrome. The design of follow-up interventions should be proactive, holistic, preventive and patient-centred [21], and gathering data about the population is essential as a first step towards a coordinated service delivery mode.

### 4.2. Population Data

Decisions about the needs of the wider demographic and socially deprived populations were essential, and the evaluation described the discrete population sub-groups for leaders to consider in their planning. Whilst the numbers were relatively small, the data demonstrate a diversity in population-level needs that would not have been evident without the visual tools. The existing capacity to visualise data in the NHS remains quite limited [22], and this paper is a case example of how the population data were achieved through shared knowledge and a commitment to achieving a new understanding to fit with the challenge of the pandemic emergency. Given the real-world nature of the project, the patient data were collected in service and transferred to the university for analysis, and whilst the data were always secure, results are in some places incomplete. However, the interpretation of patterns and trends can be drawn despite these limitations.

### 4.3. Data Visualisation

The collation, processing and visualisation of (sometimes disparate) health records can be of significant value, particularly in the chaotic early stages of a new pandemic. Modern data visualisation techniques and technologies allow practitioners without specific experience in data analytics to make sense of complex and multi-dimensional datasets [23]. The challenges associated with developing effective visualisation systems can often be organisational, connecting multiple scattered data sources into a single coherent dataset. In this project, a static data visualisation method was presented to convey a specific message to leaders and practitioners. To tackle the problem of complex decision problems, decision-makers can rely on external sources of information that will help them acquire the necessary insights into the problem. Collaboration is key to enabling effective gathering and sharing of data, and the analysis was enabled by the shared commitment to link and share data in new ways. The next step would be to create highly interactive ‘platforms’ in which the practitioner is able to present and filter the information themselves, simply and intuitively. This vastly increases the scope of possible insight and allows practitioners to pose their own questions quickly. The business intelligence function in this trust has now taken up the routine inclusion of IMD to code all patient activity, thus enabling the understanding of household deprivation as a factor in all care planning decisions. The use of Geographical Information System Mapping (GIS, which was touched upon in this project) could further enhance understanding.

### 4.4. Strengths and Limitations

The challenges encountered within this evaluation were common to others where the services are under pressure to perform but require better data management arrangements to ensure that new integrated services can be envisaged. The governance arrangements during this period facilitated the safe and appropriate use of the data to aid in the understanding of a new disease process. This enabled the communication of the patient experience to healthcare staff and leaders. Whilst it was, at times, difficult to overcome data limitations (i.e., problems with linkage and triage), the collaboration between academics and clinical leaders focused on the long-term quality of life of discharged patients [24]. Presenting data such as IMD on a map of the region gives contextual information which can help in decision making, making complex situations appear more approachable and manageable. However, as visualisations become more complex and nuanced, they become more open to individual interpretation [25], demonstrating the need to involve as many stakeholders as possible to discuss and agree on the meaning of complex datasets, with visualisations helping to broaden access to them. To enable practitioners to interact with the data and ultimately implement practices where the associated data are frequently updated, cleaned and verified, there is a need to build capacity. The most important outcome of this process was the novel insights provided to managers and frontline staff who had not previously considered health inequalities.

Despite some limitations in relation to data processing, it was an important development to report in the light of the current policy in integrated care systems in order to address inequalities in healthcare provision [26]. The outcomes of the quality-of-life data were available only for relatively small numbers, particularly when broken down into demographic subgroups. However, the data suggest that the follow-up support should be focused on some of the seldom-heard populations, such as older men who wanted to return to their ‘usual activity’ that is consistent with other findings associated with underserved groups. Further work in this domain has now taken place, with the co-production of services in underserved, more deprived populations [27].

## 5. Conclusions

This study is a pragmatic case example of an evaluation of hospital data, demonstrating how data linkage and analysis during the pandemic enabled data visualisation and shared knowledge about the population of discharged patients’ experiences. By identifying the population and mapping to the IMD, it was possible to show that over half of discharged patients were from deprived communities and that there was a high proportion of older adults in this first cohort. Of those who were offered triage for the purpose of planning their follow-on care, it was also possible to identify a perceived reduction in quality of life for many after a ‘COVID admission’. It was important to focus on patient-reported experience from the triage, a process that highlighted the psychological distress and pain that many patients reported alongside usual activities and mobility. The ethnically diverse or mixed-ethnicity groups that were admitted tended to be younger and were not in the most deprived groups. The segmenting of different populations within the data presentation brought about a realisation that medical, social and psychological factors were important to the management of COVID at this early stage of the pandemic. Several limitations are associated with working in a pragmatic way with multiple teams and datasets, but the overall outcome was useful as a demonstration of how population management knowledge could be used to plan further services.

## Figures and Tables

**Table 1:** Demographics of the 1411 patients and each subsequent sub-cohort.

	Column 1	Column 2	Column 3	Column 4
Variable	Entire cohort (*n* = 1425), *n* (% of total)	Eligible for triage (*n* = 666), *n* (% of cohort)	Started triage (*n* = 196), *n* (% eligible)	Completed triage (*n* = 167), *n* (% completed triage)
Ethnic Category ^1^				
Any mention of Asian	92 (6.5%)	68 (73.9%)	13 (19.1%)	11 (84.6%)
Any mention of black	77 (5.4%)	58 (75.3%)	14 (24.1%)	12 (85.7%)
Only white listed	1094 (76.8%)	464 (42.4%)	148 (32.0%)	125 (84.4%)
Mixed/unknown	162 (11.4%)	76 (46.9%)	21 (27.6%)	19 (90.5%)
Deprivation Pentile				
Most	638 (44.8%)	347 (54.4%)	89 (25.6%)	74 (83.1%)
2nd	168 (11.8%)	67 (39.9%)	22 (32.8%)	18 (81.8%)
3rd	218 (15.3%)	96 (44.0%)	21 (21.9%)	17 (81.0%)
4th	161 (11.3%)	74 (46.0%)	28 (37.8%)	26 (92.9%)
Least	155 (10.9%)	82 (52.9%)	28 (34.1%)	25 (89.3%)
Missing (5)	85 (6.0%)	(6)	(6)	
Gender				
Female	654 (45.9%)	315 (48.2%)	87 (27.6%)	77 (88.5%)
Male	771 (54.1%)	351 (45.5%)	109 (31.1%)	90 (82.6%)
Age Group				
17–44	307 (21.5%)	211 (68.7%)	45 (21.3%)	42 (93.3%)
55–74	438 (30.7%)	236 (53.9%)	78 (33.1%)	70 (89.7%)
75+	680 (47.7%)	219 (32.2%)	73 (33.3%)	55 (75.3%)
Patient died before or after discharge	395 (27.7%)	N/A	N/A	N/A
Care Home ^2^		N/A	N/A	N/A
Admitted from a care home	230 (16.1%)			
Discharged to a care home	258 (18.1%)			
Missing	85 (6.0%)			
1 + spells in ITU	150 (10.5%)	99 (66.0%)	29 (29.3%)	28 (96.6%)
Number of Inpatient Stays				
1	1235 (86.7%)	579 (46.9%)	165 (28.5%)	143 (86.7%)
2	156 (10.9%)	70 (44.9%)	26 (37.1%)	22 (84.6%)
3–5	34 (2.4%)	17 (50.0%)	5 (29.4%)	2 (40.0%)
Longest Stay in Hospital ^3^				
0–2 days ^4^	295 (20.7%)	143 (48.5%)	12 (8.4%)	10 (83.3%)
3–7 days	372 (26.1%)	211 (56.7%)	63 (29.9%)	55 (87.3%)
8–16 days	362 (25.4%)	158 (43.6%)	78 (49.4%)	65 (83.3%)
17 + days	396 (27.8%)	154 (38.9%)	43 (27.9%)	37 (86.0%)
Referred to community services	765 (53.7%)	457 (68.6%)	121 (26.5%)	102 (84.3%)
Had a new OP appointment	814 (57.1%)	475 (58.4%)	138 (29.1%)	119 (86.2%)

^1^ Given that BAME groups are reported to be at a greater risk, the supplied ethnic categories were coded in a different way—see main text. ^2^ Residents outside the region were not eligible, but they were inadvertently included in the early days. ^3^ If there were multiple stays, the longest one was selected. ^4^ Initially, patients had to be admitted to be tested and confirmed for COVID-19.

## Data Availability

Data reported in this paper are for the purpose of describing the process and learning and are not available.

## Appendix A. Classification of the Ethnic Diversity within the Dataset

Conventional coding was used in the previous report while Ethcat2 was used in the sub-analyses in this report. Neither classification is satisfactory, but small numbers in any version hamper analyses as numbers decline with each subdivision of the cohort.

**Original List**	N	**Conventional Coding**			**Alternative (Ethcat2)**		
		**Code**	**Label**	**Count**	**Code**	**Label**	**Count**
Bangladeshi	1	1	Asian			Any mention of Asian	
Any Other Asian Background	16	1	Asian	89	1	Any mention of Asian	92
Chinese	4	1	Asian		1	Any mention of Asian	
Indian	8	1	Asian		1	Any mention of Asian	
Mixed White and Asian	3	3	Mixed		1	Any mention of Asian	
Pakistani	60	1	Asian		1	Any mention of Asian	
Any Other Black Background	17	2	Black	74	2	Any mention of Black	77
Black African	32	2	Black		2	Any mention of Black	
Black Caribbean	25	2	Black		2	Any mention of Black	
Mixed White and Black African	1	3	Mixed		2	Any mention of Black	
Mixed White and Black Caribbean	2	3	Mixed		2	Any mention of Black	
Any Other White Background	19	4	White	1094	3	White only	1094
White British	1069	4	White		3	White only	
White Irish	6	4	White		3	White only	
Any Other Ethnic Group	40	3	Mixed	55	4	Unknown/mixed	162
Any Other Mixed Background	9	3	Mixed		4	Unknown/mixed	
Not Known	38	5	Missing	113	4	Unknown/mixed	
Not Stated	75	5	Missing		4	Unknown/mixed	
